# Supplementary material for: Effects of a Novel Applet-Based Personalized Dietary Intervention on Dietary Intakes: A Randomized Controlled Trial in a Real-World Scenario
Source: Nutrients. 2024 Feb 19;16(4):565. doi: 10.3390/nu16040565 (PMC10892066; doi:10.3390/nu16040565)
Supplement: Supplementary file 1 [file nutrients-16-00565-s001.zip › nutrients-2863567-supplementary.pdf]

Table S1. Comparison of baseline characteristics between the participants included and excluded.

	ALL	Participants included	Participants excluded	P value
n	177	153	24	
Age, years, mean (SD)	33.1 (7.8)	32.7 (7.5)	35.7 (9.2)	0.081
Sex, n (%)				
Female	101 (57.1)	97 (63.4)	4 (16.7)	<0.001
Male	76 (42.9)	56 (36.6)	20 (83.3)	
Smoking status, n (%)				
Non-smoker	150 (84.7)	136 (88.9)	14 (58.3)	<0.001
Ex-smoker	14 (7.9)	11 (7.2)	3 (12.5)	
current Smoker	13 (7.3)	6 (3.9)	7 (29.2)	
Alcohol consumption, n (%)				
Lifetime abstainer	72 (40.7)	67 (43.8)	5 (20.8)	0.001
Non-heavy drinkers	77 (43.5)	68 (44.4)	9 (37.5)	
Heavy drinkers	28 (15.8)	18 (11.8)	10 (41.7)	
Physical activity level, n (%)				
Low	154 (87.0)	134 (87.6)	20 (83.3)	0.314
Moderate	21 (11.9)	18 (11.8)	3 (12.5)	
High	2 (1.1)	1 (0.7)	1 (4.2)	
Intentional physical exercise, n (%)				
No	135 (76.3)	118 (77.1)	17 (70.8)	0.678
Yes	42 (23.7)	35 (22.9)	7 (29.2)	
Enrollment sequence, n (%)				
September	23 (13.0)	23 (15.0)	0 (0.0)	0.118
October	101 (57.1)	86 (56.2)	15 (62.5)	
November	53 (29.9)	44 (28.8)	9 (37.5)	

Table S2. Effects of the intervention on average weekly dietary changes of lunch: Sensitivity analysis.

	Time × Group	
	β	p
Food group, g/meal		
Plant foods	4.52	0.016
Cereals and Tubers	0.16	0.770
Vegetables & Fruits	4.41	0.015
Soybeans and soybean products	0.03	0.878
Animal foods	-1.86	0.093
Livestock and poultry meat	-1.99	0.061
Aquatic products	0.48	0.560
Eggs and egg products	-0.68	0.130
Animal / plant food ratio	-0.03	0.002
Energy, kcal/meal	-6.32	0.049
Percentages of energy intake from fat	0.00	0.575
Nutrients		
Protein, g/meal	-0.29	0.111
Fat, g/meal	-0.39	0.077
Carbohydrate, g/meal	-0.05	0.840
Cholesterol, mg/meal	-5.21	0.027
Sodium, mg /meal	-10.33	0.477
Calcium, mg/meal	1.69	0.347
Iron, mg/meal	-0.06	0.266
Zinc, mg/meal	0.00	0.895
Vitamin C, mg/meal	0.34	0.348

NOTE: The significant interaction item (Time × Group) would indicate overall trend differences between the both groups; Model were adjusted for enrollment sequence, the dependent variables' baseline value, age, sex, baseline BMI, physical activity level, intentional physical exercise, smoking status and alcohol consumption; Model excluded participants who changed exercise routine or lunch dietary intake as a proportion of total daily dietary intake (n=20) at follow-up period; β value represented average weekly change in dietary intakes.

Table S3. Effects of the intervention on average monthly changes of anthropometric indicators: Sensitivity analysis.

	Time × Group	
	β	p
Body weight, kg	-0.449721	0.0035
BMI, kg/m^2	-0.257566	0.0244
Blood pressure, mmHg		
Systolic pressure	-0.228722	0.8877
Diastolic pressure	1.199244	0.1984
Body composition		
Percentage of body fat, %	-0.345244	0.4485
Percentage of torso fat, %	-0.050636	0.9239
Visceral fat index	-0.192772	0.4045

NOTE: The significant interaction item (Time × Group) would indicate overall trend differences between the both groups; Model were adjusted for enrollment sequence, the dependent variables' baseline value, age, sex, physical activity level, intentional physical exercise, smoking status and alcohol consumption; Model excluded participants who changed exercise routine or lunch dietary intake as a proportion of total daily dietary intake (n=18) at follow-up period; β value represented average monthly change in anthropometric indicators.
